# Behavioral Tracking Studies in Online Gambling: A Note on Delfabbro et al. (2023)

**DOI:** 10.1007/s10899-023-10271-6

**Published:** 2023-12-07

**Authors:** Mark D. Griffiths

**Affiliations:** International Gaming Research Unit, Psychology Department, School of Social Sciences, Nottingham Trent University, 50 Shakespeare Street, Nottingham, NG1 4FQ UK

Dear Dr. Grant,

I was interested to read the paper ‘Behavioural tracking and profiling studies involving objective data derived from online operators: A review of the evidence’ by Delfabbro et al. (2023). As someone who has been publishing papers for over a decade using data provided by the gambling industry, I was pleased to see that 20 studies that I co-authored were among the 58 outputs identified in the review (56 peer-reviewed papers and two *“major reports”* [p.4]). However, many studies were missing from the review.

The paper’s aim was to systematically review what is *“currently known from 15 years of studies involving the analysis of objective behavioural data sourced from gambling operators”* (p. 3) up until December 2022. The authors also noted that for inclusion in the review, the study *“had to involve objective online behavioural data in at least some part of the research rather than self-reported attitudes or knowledge relating to online gambling”* (p.4). The review also noted that the search for studies had used the *Scopus* database and that *“further publications were identified through some earlier published reviews; through inspection of secondary sources cited in the most recent (2022) papers”* (p.4).

Given that the aims of the study were clear and that the only inclusion criterion was that the study had to include online behavioral tracking data, I was somewhat surprised that 14 studies that I co-authored and were published before December 2022 were not in the review (i.e., Auer & Griffiths, 2014, 2015a, 2018, 2020, 2022a, 2022b; Auer et al., 2018, 2019, 2023 [this paper was first published online on May 29, 2020]; Chagas et al., 2022; Hopfgartner et al., 2021; Leino et al., 2015, 2017; Sagoe et al., 2018). Three of these studies admittedly included video lottery terminal (VLT) player card data provided by the Norwegian gambling operator *Norsk Tipping* (Leino et al., 2015, 2017; Sagoe et al., 2018), but all three studies arguably fitted the remit of the review and *Norsk Tipping* is an online (as well as offline) gambling operator. However, the other 11 studies all clearly fitted the inclusion criterion. Moreover, most of these studies had been cited in the other papers that were included in the review (especially those that I co-authored).

It should also be noted that one of the studies that appears to be missing (i.e., Auer & Griffiths, 2015a) may have been in the review but was mis-referenced (i.e., Auer & Griffiths, 2015b) although both of these papers contained behavioral tracking data and both should have been included based on the review’s remit.

It was also unclear why the two *“major reports”* in the gray literature by *Price-Waterhouse *Coopers (2017) and Forrest and McHale (2022) were included and not others. Both these studies were published by *GambleAware* but other major reports by *GambleAware* using behavioral tracking data have been published (such as those by the Behavioural Insights Team [2021a, 2021b]) but none of these were cited.

## Data Availability

Not applicable.

## References

[CR1] Auer, M., & Griffiths, M. D. (2014). Personalised feedback in the promotion of responsible gambling: A brief overview. *Responsible Gambling Review,**1*, 27–36.

[CR2] Auer, M., & Griffiths, M. D. (2015a). Testing normative and self-appraisal feedback in an online slot-machine pop-up message in a real-world setting. *Frontiers in Psychology,**6*, 339. 25852630 10.3389/fpsyg.2015.00339 PMC4369874

[CR3] Auer, M., & Griffiths, M. D. (2015b). Theoretical loss and gambling intensity (revisited): A response to Braverman et al. (2013). *Journal of Gambling Studies, 31*, 921–931. 10.1007/s10899-014-9463-4 24760196

[CR4] Auer, M., & Griffiths, M. D. (2018). Cognitive dissonance, personalized feedback, and online gambling behavior: An exploratory study using objective tracking data and subjective self-report. *International Journal of Mental Health and Addiction,**16*, 631–641. 29904326 10.1007/s11469-017-9808-1 PMC5986838

[CR5] Auer, M., & Griffiths, M. D. (2020). The use of personalized messages on wagering behavior of Swedish online gamblers: An empirical study. *Computers in Human Behavior,**110*, 106402. 10.1016/j.chb.2020.106402

[CR6] Auer, M., & Griffiths, M. D. (2022a). Gambling before and during the COVID-19 pandemic among online casino gamblers: An empirical study using behavioral tracking data. *International Journal of Mental Health and Addiction,**20*, 1722–1732. 33551689 10.1007/s11469-020-00462-2 PMC7852466

[CR7] Auer, M., & Griffiths, M. D. (2022b). Predicting limit-setting behavior of gamblers using machine learning algorithms: A real world study of Norwegian gamblers using account data. *International Journal of Mental Health and Addictions,**20*, 771–788. 10.1007/s11469-019-00166-2

[CR8] Auer, M., Hopfgartner, N., & Griffiths, M. D. (2018). The effect of loss-limit reminders on gambling behavior: A real world study of Norwegian gamblers. *Journal of Behavioral Addictions,**7*(4), 1056–1067. 30418076 10.1556/2006.7.2018.106 PMC6376395

[CR9] Auer, M., Hopfgartner, N., & Griffiths, M. D. (2019). The effects of a mandatory play break on subsequent gambling among Norwegian video lottery terminal players. *Journal of Behavioral Addictions,**8*(3), 522–529. 31537088 10.1556/2006.8.2019.51 PMC7044625

[CR10] Auer, M., Malischnig, D., & Griffiths, M. D. (2023). Gambling before and during the COVID-19 pandemic among European regular sports bettors: An empirical study using behavioral tracking data. *International Journal of Mental Health and Addiction,**21*, 20–27. 32837423 10.1007/s11469-020-00327-8 PMC7259431

[CR11] Behavioural Insights Team. (2021a). *Applying behavioural insights to design better safer gambling tools. Part 1: Anchoring: Commitment devices.* GambleAware.

[CR12] Behavioural Insights Team (2021b). *Applying behavioural insights to design better safer gambling tools. Part 2: Commitment devices.* GambleAware.

[CR13] Chagas, B. T., Gomes, J. F. S., & Griffiths, M. D. (2022). Consumer profile segmentation in online lottery gambling utilizing behavioral tracking data from the Portuguese national lottery. *Journal of Gambling Studies,**38*, 917–939. 34518986 10.1007/s10899-021-10072-9 PMC8437336

[CR14] Delfabbro, P., Parke, J., & Catania, M. (2023). Behavioural tracking and profiling studies involving objective data derived from online operators: A review of the evidence. *Journal of Gambling Studies*. 10.1007/s10899-023-10247-6 37634166 10.1007/s10899-023-10247-6 PMC11272745

[CR15] Forrest, D. & McHale, I. (2022). In I. McHale (Ed.), *Patterns of play* (Technical report., 2 vol.). GambleAware.

[CR16] Hopfgartner, N., Santos, T., Auer, M., Griffiths, M. D., & Helic, D. (2021). Social facilitation among gamblers: A large-scale study using account-based data. *Proceedings of the International AAAI Conference on Web and Social Media,**15*(1), 185–195. 10.1609/icwsm.v15i1.18052

[CR17] Leino, T., Sagoe, D., Griffiths, M. D., Mentzoni, R. A., Pallesen, S., & Molde, H. (2017). Gambling behavior in alcohol-serving and non-alcohol-serving venues: A study of electronic gaming machine players using account records. *Addiction Research and Theory,**25*, 201–207. 10.1080/16066359.2017.1288806

[CR18] Leino, T., Torsheim, T., Blaszczynski, A., Griffiths, M. D., Mentzoni, R., Pallesen, S., & Molde, H. (2015). The relationship between structural characteristics and gambling behavior: A population level study. *Journal of Gambling Studies,**31*, 1297–1315. 24923625 10.1007/s10899-014-9477-y PMC4651975

[CR19] Price-Waterhouse Coopers. (2017). *Remote gambling research. Interim report on phase II.* GambleAware.

[CR20] Sagoe, D., Pallesen, S., Griffiths, M.D., Mentzoni, R.A. & Leino, T. (2018). Does individual gambling behavior vary across gambling venues with differing numbers of terminals? An empirical real-world study using player account data. An empirical real world study. *Frontiers in Psychology, 9,* 158. 10.3389/fpsyg.2018.00158 PMC5820341 29503626

